# A pilot study on investigating the role of *Salvia miltiorrhiza* in fetal growth restriction

**DOI:** 10.1042/BSR20201222

**Published:** 2020-06-03

**Authors:** Fanghua Shen, Hongdao Lv, Yun Shi, Sandy Lau, Fang Guo, Qi Chen

**Affiliations:** 1 Department of Obstetrics and Gynaecology, Suzhou Ninth People’s Hospital, Suzhou, China; 2 Department of Obstetrics and Gynaecology, The University of Auckland, Auckland, New Zealand; 3 The Hospital of Obstetrics and Gynaecology, Fudan University, Shanghai, China

**Keywords:** abdominal circumference, biparietal diameter, birthweight, FGR, Salvia miltiorrhiza

## Abstract

To date there is no effective treatment for pregnancies complicated by fetal growth restriction (FGR). *Salvia miltiorrhiza*, a traditional Chinese herb has been shown to promote blood flow and improve microcirculatory disturbance. In this pilot study, we evaluated whether *S. miltiorrhiza* can potentially become a possible therapy for FGR. Nineteen pregnant women with FGR were treated with *S. miltiorrhiza* and ATP supplementation for an average of 7 days, and 17 cases received ATP supplementation as controls. The estimated fetal weights (EFWs) were measured by ultrasound after treatment, and the birthweights were recorded after birth. After treatment with *S. miltiorrhiza*, 7 (37%) FGR cases showed an increase in EFW to above the 10th percentile, compared with 4 (23%) FGR cases in controls (odds ratio: 1.896, 95% confidence limits (CLs): 0.44–8.144). At delivery, 10 (53%) FGR cases in the treatment group delivered babies with a birthweight above the 10th percentile, compared with 6 (35%) FGR cases in the control group (odds ratio: 2.037, 95% CL: 0.532–7.793); 80 or 64% FGR cases in the treatment group showed an increase in fetal abdominal circumference (AC) or biparietal diameter (BPD) above the 10th percentile before delivery. While 44 or 30% FGR cases in the control group showed an increase in AC or BPD. No improvement of head circumference (HC) or femur length (FL) was seen. These pilot data suggest the need for multicenter randomized clinical trials on the potential of *S. miltiorrhiza* to improve perinatal outcome in pregnant women complicated by FGR.

## Introduction

Fetal growth restriction (FGR) refers to a condition in which a fetus is unable to achieve its genetically determined potential size, because of genetic or environmental factors. The origin of these factors may be fetal, placental or maternal, with significant overlap among these entities. FGR affects 3–10% of pregnancies worldwide and is a major cause for preterm birth, stillbirth in late pregnancy [1] and neonatal morbidity, and is a cause of neurological and cardiovascular diseases in adulthood [2,3]. However due to the poor understanding of the etiology of FGR, the optimal treatment for FGR is still limited and has some risks [4]. Currently, treatment for FGR is dependent on the gestational age of the fetus at presentation, including early or immediate delivery for fetal well-being, improved nutrition for maternal weight gain and bed rest in order to improve blood flow to the baby. However, bed rest has not been found to improve perinatal outcomes and therefore is not typically recommended [5].

*Salvia miltiorrhiza,* also known as Danshen in Chinese traditional medicine, is extracted from a perennial plant in the genus *Salvia* of the mint family, Lamicaeae [6]. *S. miltiorrhiza* has been widely used in treatment with vascular diseases including cardiovascular disease, atherosclerosis, thrombosis and cerebral infarction with ischemic condition in China and other Asian countries for several decades [7–10]. *S. miltiorrhiza* contains the lipid-soluble tanshinone I (Tan I), tanshinone IIA (Tan IIA), cryptotanshinnoe and dihydrotan-shinone as well as danshensu and salvianolic acid B [9]. Danshensu and salvianolic acid B have been shown to have antioxidative properties by reducing inflammation and improving angiogenesis (reviewed in [11]). In addition, the compound of tanshinones, dihydrotan-shinone and cryptotanshinnoe have been shown to inhibit the production of interleukin (IL) 12 (IL-12) or IL-6 or THF-a induced by lipopolysaccharide (LPS) in activated macrophages [12,13]. These studies suggested that *S. miltiorrhiza* can promote blood flow to resolve blood stasis [14] by inhibiting platelet aggregation and promoting fibrinolysis [15,16].

When blood flow to the uterus is decreased, the blood flow through the placenta that reaches the fetus is also reduced (poor uteroplacental blood flow). Subsequently, delivery of oxygen and nutrients essential for the growth of the fetus is also reduced, which occurs in cases of FGR. Current evidence does not support the notion that antenatal identification of FGR and conservative management alone is enough to improve fetal outcomes. In some cases of FGR, pharmacological intervention to improve placental function and maternal blood flow across the placenta may be required to improve perinatal outcomes [17]. In addition, increased inflammation is associated with the causes of FGR by inhibition spiral artery remodeling [18,19]. Due to the pharmacological effects of *S. miltiorrhiza* on improving blood flow and anti-inflammation, in the present study, we performed a pilot retrospective study to investigate the effect of treatment with *S. miltiorrhiza* on improving the birthweight in pregnancies complicated by FGR. We hypothesized that treatment with *S. miltiorrhiza* can improve the birthweight in pregnant women complicated with FGR. The main outcomes for the present study were to investigate: (1) the proportion of FGR cases whose estimated fetal weight (EFW) were increased to above 10th percentile after treatment, (2) the proportion of FGR cases whose birthweight were above 10th percentile at delivery, and (3) the proportion of FGR cases whose fetal biparietal diameter (BPD), abdominal circumference (AC), HC and femur length (FL) were increased above 10th percentile age [20] after treatment, and before delivery.

## Materials and methods

This investigation conforms to the principles outlined in the Declaration of Helsinki. The present study was approved by the Ethics Committee of Ninth Hospital of Suzhou, Jiangsu Province of China (Reference 2016-007). All subjects provided the written consent form.

### Study population

During January 2017 to January 2018, a total number of 19 FGR cases received the treatment with *S. miltiorrhiza* solution (20 g *S. miltiorrhiza* in 500 ml of 5% glucose solution, Chiatal Qingchunbao Pharmaceutical Co. Ltd, China) plus ATP supplementation (ATP 40 mg and CoA 200 mg in 500 ml of 5% glucose solution) via an intravenous drip, once a day in our hospital. The dose of *S. miltiorrhiza* used in the present study was followed on from a previous study [21]. In comparison, 19 FGR cases who received the treatment with a solution of ATP supplementation only were included as a control group, however, two cases withdrew from the study for personal reasons. The gestational age at diagnosis for all FGR cases were over 28 weeks. Pregnant women with preeclampsia, gestational diabetes mellitus, other cardiovascular diseases or history of FGR in a previous pregnancy, were excluded.

After 7 consecutive days of treatment with *S. miltiorrhiza*, the fetal growth parameters were assessed with Doppler ultrasound at which point the Obstetrician decided whether another series of treatment of 7 days was required. One of the main criteria for the decision to extend treatment was the estimation of fetal weight. The median days for treatment were 7 days (range: from 7 to 21 days). None of the women in the control or treatment groups developed preeclampsia later during the period of study. Decision to deliver was based on the standard clinical assessment in our hospital.

Data on these 36 FGR cases including maternal age, parity, gravida, gestational age at diagnosis, gestational age at delivery, birthweight, Apgar score at 1 and 5 min were collected as well as the duration of treatment. Prenatal Doppler ultrasound data including BPD, AC, HC and FL, as well as umbilical artery systolic/diastolic (S/D) ratio were collected at the time of diagnosis, after treatment and again 1 day before delivery. Estimation of fetal weight (EFW) was calculated by using BPD, HC, AC and FL (EFW = 1.3596-0.00386AC*FL+0.0064HC+.00061BPD*AC+0.0424AC+0.174FL) as published in a previous study, after adjusting the gestational age [22]. The percentiles for EFW or BPD, AC, HC, FL or birthweight were determined using the WHO fetal growth chart based on the gestational age [20]. The duration of BPD, AC, HC and FL analysis was at least 30 days. All mothers and infants were followed up until 42 days postpartum.

FGR is defined by an EFW below the 10th percentile for its gestational age [23–25]. Qualified and experienced technicians who were blinded to the treatment and control groups performed Doppler ultrasound for all measurements.

### Statistical analysis

Data were presented as median and range or percentage as appropriate. The statistical differences in maternal age, gestational week at diagnosis, gestational week at delivery and duration of treatment were assessed by the Mann–Whitney U-test using the Prism software package. Statistical differences in parity and modes of birth were assessed by Chi-square test using the Prism software package. The analysis in EFW and AC or BPD, or head circumference (HC) was assessed by odds ratio and 95% confidence limits (CLs) using Fisher’s exact test by OpenEpi software. A *P*-value of <0.05 was considered significant.

## Results

During the study period, 36 FGR cases were included and clinical characteristics of study cohort are summarized in Table 1. The median days for receiving treatment were 7 days (range: from 7 to 21 days) in both groups. There was no stillbirth and no infants with Apgar score below 7 at 1 or 5 min in both groups. There was no statistical difference in rates of cesarean sections between two groups.

**Table 1:** Clinical parameters of study cohort

	Treatment group (*n*=19)	Control group (*n*=17)	*P*-value (Fisher’s exact test)
Maternal age (year, median/range)	28 (20–37)	26 (23–34)	*P*=0.445
Gestational age at diagnosis (week, median/range)	32^+2^ (28–36^+6^)	34^+3^ (29–36^+6^)	*P*=0.028
Gestational age at delivery (week, median/range)	38^+3^ (36–41^+6^)	38^+3^ (36–40^+6^)	*P*=0.735
Nulliparous (number, %)	12 (64%)	9 (53%)	*P*=0.736
Days of treatment (median/range)	7 (7–21)	7 (7–21)	*P*=0.357
Cesarean section (number, %)	9 (47%)	6 (35%)	*P*=0.516

We first analyzed the EFW after treatment and before delivery, and birthweight at delivery. Seven out of 19 (37%) FGR cases in the treatment group showed an increase in the EFW to the above 10th percentile after treatment. In contrast, only 4 out of 17 (23%) of FGR cases in the control group showed an improvement of the EFW to above the 10th percentile. The odds ratio of an increase in EFW to the above 10th percentile in FGR cases who received treatment was 1.896 (95% CL: 0.44–8.144, Table 2), compared with FGR cases in the control group. At one day before delivery, 8 out of 19 (42%) FGR cases in the treatment group showed an increase in the EFW to the above 10th percentile after treatment. In contrast, only 5 out of 17 (29%) of FGR cases in the control group showed an improvement of the EFW to the above 10th percentile. The odds ratio of an increase in EFW to the above 10th percentile in FGR cases in the treatment group was 1.745 (95% CL: 0.437–6.971, Table 2), compared with FGR cases in the control group. At birth, 10 of 19 (53%) babies from the treatment group presented with a birthweight above the 10th percentile at delivery, but only 6 out of 17 (35%) babies in the control group presented with a birthweight above the 10th percentile. The odds ratio of a birthweight above the 10th percentile in FGR cases in the treatment group was 2.037 (95% CL: 0.532–7.793, Table 2), compared with FGR cases in the control group.

**Table 2:** EFW after treatment and birthweight at delivery

	Treatment group (*n*=19)	Control group (*n*=17)	Odds ratio (95% CL) (Fisher’s exact test)
EFW above 10th percentile after treatment (number, %)	7 (37%)	4 (23%)	1.896 (0.44–8.144)
EFW above 10th percentile before delivery (number, %)	8 (42%)	5 (29%)	1.745 (0.437, 6.971)
Birthweight above 10th percentile (number, %)	10 (53%)	6 (35%)	2.037 (0.532–7.793)

We next analyzed fetal AC, BPD, HC and FL after treatment and 1 day before delivery. Prior to treatment, the number of FGR cases with an AC or BPD or HC below the 10th percentile at time of diagnosis were 10 (57%) or 14 (74%) or 15 (79%) in the treatment group. In the control group, the number of FGR cases with an AC or BPD or HC below 10th percentile at time of diagnosis were 9 (53%) or 10 (59%) or 15 (88%) or 9 (53%).

After *S. miltiorrhiza* treatment, 6 (60%) or 7 (50%) or 4 (27%) FGR cases whose original AC or BPD or HC was below the 10th percentile showed an increase in the AC or BPD or HC to above 10th percentile. In contrast, in the control group, 4 (44%) or 2 (20%) or 3 (20%) FGR cases showed an increase in the AC or BPD or HC above the 10th percentile (Supplementary Tables S1–S3, respectively). The odds ratio of an increase in AC or BPD, or HC to above 10th percentile was 1.875 (95% CL: 0.302–11.62), or 4.000 (95% CL: 0.588–22.66), or 1.455 (95% CL: 0.319–6.728), respectively in the treatment group, compared with the control group.

At one day before delivery, 8 (80%) or 9 (64%) or 9 (60%) FGR cases in the treatment group showed an increase in the AC, or BPD, or HC above the 10th percentile. However, in control group, 4 (44%), or 3 (30%), or 6 (40%) FGR cases showed an increase in the AC, or BPD, or HC to above 10th percentile (Supplementary Tables S1–S3). The odds ratio of AC, or BPD, or HC above 10th percentile in treatment group was 5.000 (95% CL: 0.655–38.15) or 4.2 (95% CL: 0.715–19.14) or 2.25 (95% CL: 0.561–10.22), respectively, compared with the control group.

Prior to treatment, there were six FGR cases (26%) whose FL was below 10th percentile at the time of diagnosis. However, there was no improvement on FL, either after treatment or 1 day before delivery (Supplementary Table S4). There were also no changes of umbilical artery S/D ratio after treatment or 1 day before delivery in the treatment and control group (data not shown).

## Discussion

In this pilot retrospective study, we found that after treatment with *S. miltiorrhiza* for a median of 7 days (range: from 7 to 21 days), almost twice as many FGR cases in the treatment group showed an increase in the EFW to above the 10th percentile, compared with FGR cases in the control group. We also found that at delivery, 53% of FGR cases in the treatment had babies whose birthweight was above the 10th percentile. In comparison, only 35% of FGR cases in the control group gave birth to babies who had birthweights above 10th percentile at delivery. The odds ratio of FGR cases who gave birth to babies with a birthweight above 10th percentile was 2.037 (95% CL: 0.532–7.793) if they received the treatment, compared with FGR cases who did not receive *S. miltiorrhiza* treatment.

In addition, after treatment with *S. miltiorrhiza,* 60 or 50% of FGR cases in the treatment group showed an increase in the AC or BPD to above the 10th percentile, compared with FGR cases (44 or 20%) in the control group. Furthermore, at 1 day before delivery, we also found that 80 or 64% FGR cases who received *S. miltiorrhiza* treatment during pregnancy had babies whose AC or BPD were above the 10th percentile. In comparison, in the control group, 44 or 30% of FGR show improvement with the AC or BPD above the 10th percentile. The odds ratio of FGR cases whose babies with an AC or BPD above 10th percentile at 1 day before delivery was 5.000 (95% CL: 0.655–38.15) or 4.200 (95% CL: 0.715–19.14) if they received the treatment, compared with FGR cases who did not receive *S. miltiorrhiza* treatment.

However, in our study we only see a small proportion of FGR cases having improvement on HC or no FGR cases had improvement on FL after treatment, compared with the control groups.

Currently there is no effective treatment for FGR. Balancing the risks of intrauterine demise (stillbirths) and prematurity by early delivery is a subjective decision posing a challenge for obstetricians around the world. FGR is associated with hypercoagulation and a dysfunctional microcirculation [26] and this may result in a reduced blood flow to the fetus. Therefore, hypercoagulation may be one of the targets for pharmacological intervention after the diagnosis of FGR to improve perinatal outcomes. Several evidences have suggested that *S. miltiorrhiza*, a Chinese herb can promote improved microcirculation and improve angiogenesis, and thereby improving blood circulation in the uterus and placenta. A previous study by a Chinese group also reported that the treatment with *S. miltiorrhiza* can improve the average birthweight of babies who were born in FGR cases [21]. Our preliminary data from our pilot retrospective study further suggest that treatment with *S. miltiorrhiza* at the time of diagnosis increases the EFW, AC, BPD and birthweight in pregnancies complicated by FGR.

In this retrospective study, although data on neonatal outcomes including long term follow-up are limited, there were no cases of stillbirth and no cases of infant’s Apgar score below 7 at 1 or 5 min at delivery time in both groups. In Trial of Randomized Umbilical and Fetal Flow in Europe (TRUFFLE) study, which was a conservative study [27], the birthweight in FGR cases was smaller and the delivery time was earlier. The median time to delivery in the TRUFFLE study was 13 days for FGR cases without gestational hypertension, and perinatal morbidity including stillbirth were significantly related to gestational age. However, in our current pilot retrospective study we clearly showed 53% of babies in FGR cases with *S. miltiorrhiza* treatment whose birthweight above 10th percentile which was higher than untreated FGR cases. The gestational age in FGR cases at the time of onset was slightly different between our current study (28–36 weeks of gestation) and the TRUFFLE study (26–32 weeks of gestation), which calls for caution when making direct comparisons between the reported outcomes of the two studies. In addition, in comparison with another study [28], the FGR cases included in this pilot retrospective study were not severe FGR (AC below 1st percentile, EFW below 3rd percentile) and were also not early onset FGR (before 22 weeks of gestation). It is unknown whether the same pathogenic mechanisms underlie both the severe and mild forms of FGR.

*S. miltiorrhiza*, a widely used herb in traditional medicine in China and other Asian countries has been shown to reduce blood viscosity, by acting as an anticoagulant possibly through its effect on thrombocytes [15,16]. *S. miltiorrhiza* can promote improved microcirculation [29] and thereby improving blood circulation in the uterus and placenta. This consequently increases the delivery of factors essential for growth of fetuses in FGR. In addition, *S. miltiorrhiza* also showed a beneficial effect on treating pregnant women with oligohydramnios [30] and pregnancy-induced hypertension [31]. *S. miltiorrhiza,* as the first traditional Chinese medicine has completed a Phase II clinical trial and entered into a Phase III clinical trials for heart disease in the United States [7,32]. Therefore, *S. miltiorrhiza* may be a novel pharmaceutical intervention for pregnancies complicated by FGR to improve perinatal outcome in future.

In this observational study, we found there was no difference in umbilical artery S/D ratio between treatment and control group. Although we did not investigate the underlying mechanisms of *S. miltiorrhiza* on improve fetal growth in pregnancy complicated with FGR, previous studies reported that *S. miltiorrhiza* has anticoagulant and vasodilatory effect, resulting in increased blood flow [15,16,33]. These studies suggest that the effect of *S. miltiorrhiza* reported in our current study may not be due to the changes of peripheral resistance. Future studies, in particular whether *S. miltiorrhiza* enhances placental vasculature development are required, in order to better understand its putative beneficial effort.

There are some limitations in the present study. First, due to the small sample size of the FGR cases in this pilot retrospective study, we were not able to show statistically significant odds ratios for FGR cases who received the treatment with *S. miltiorrhiza* compared with FGR cases who did not. However, in this pilot retrospective study we found an increase in EFW in twice as many FGR cases who received treatment with *S. miltiorrhiza*, compared with FGR cases who did not. Second, data on neonatal outcomes including long term follow-up are limited. Third, data on pre-pregnancy BMI and weight gain were not available in our record. Due to the small sample size, we were not able to adjust the confounding factors in order to avoid causing wider confidence intervals.

## Conclusion

In this pilot retrospective study, we showed the improvement in birthweight in FGR cases who received *S. miltiorrhiza* treatment and suggest that there may be a clinical significance in improving birthweight in FGR cases after *S. miltiorrhiza* treatment. Our data suggest that *S. miltiorrhiza* may offer a new opportunity to improve perinatal outcomes in pregnancies complicated by FGR, but we recommend that our current pilot retrospective study including severe and early onset FGR should be repeated with a larger sample size by multicenters in an international collaboration in the future.

## Highlights

1. To date there is no effective treatment on FGR.
2. *S. miltiorrhiza*, a traditional Chinese herb has been widely used in treatment with vascular diseases for many years in China.
3. *S. miltiorrhiza* improved the birthweight in pregnancy complicated by FGR.

## Supplementary Material

Supplementary Tables S1-S4

## Abbreviations

AC: abdominal circumference
BPD: biparietal diameter
CL: confidence limit
EFW: estimated fetal weight
FGR: fetal growth restriction
FL: femur length
HC: head circumference
IL: interleukin
S/D: systolic/diastolic
TRUFFLE: Trial of Randomized Umbilical and Fetal Flow in Europe
