# Supplementary material for: A pilot study on investigating the role of Salvia miltiorrhiza in fetal growth restriction
Source: Biosci Rep. 2020 Jun 3;40(6):BSR20201222. doi: 10.1042/BSR20201222 (PMC7269916; doi:10.1042/BSR20201222)
Supplement: Supplementary Tables S1-S4 [file BSR-2020-1222_supp.pdf]

Supplementary Table 1: Abdominal circumference (AC) after treatment and before delivery

	AC below 10th percentile at diagnosis in treatment group (n=10)	AC below 10th percentile at diagnosis in control group (n=9)	Odds ratio (95% CL) (Fisher's exact test)
AC above 10th percentile after treatment (number, %)	6 (60%)	4 (44%)	1.875 (0.302-11.62)
AC above 10th percentile before delivery (number, %)	8 (80%)	4 (44%)	5.00 (0.655-38.15)

Supplementary Table 2: Biparietal diameter (BPD) after treatment and before delivery

	BPD below 10th percentile at diagnosis in treatment group (n=14)	BPD below 10th percentile at diagnosis in control group (n=10)	Odds ratio (95% CL) (Fisher's exact test)
BPD above 10th percentile after treatment (number, %)	7 (50%)	2 (20%)	4.000 (0.588-22.66)
BPD above 10th percentile before delivery (number, %)	9 (64%)	3 (30%)	4.20 (0.715-19.14)

Supplementary Table 3: Head circumference (HC) after treatment and before delivery

	HC below 10th percentile at diagnosis in treatment group (n=15)	HC below 10th percentile at diagnosis in control group (n=15)	Odds ratio (95% CL) (Fisher's exact test)
HC above 10th percentile after treatment (number, %)	4 (27%)	3 (20%)	1.455 (0.319-6.728)
HC above 10th percentile before delivery (number, %)	9 (60%)	6 (40%)	2.25 (0.561-10.22)

Supplementary Table 4: Femur Length (FL) after treatment and before delivery

	FL below 10th percentile at diagnosis in treatment group (n=5)	FL below 10th percentile at diagnosis in control group (n=9)	Odds ratio (95% CL)
FL above 10th percentile after treatment (number, %)	0 (0%)	1 (11%)	N/A
FL above 10th percentile before delivery (number, %)	0 (0%)	2 (22%)	N/A

N/A: not applicable
